# Timescales of methane seepage on the Norwegian margin following collapse of the Scandinavian Ice Sheet

**DOI:** 10.1038/ncomms11509

**Published:** 2016-05-11

**Authors:** Antoine Crémière, Aivo Lepland, Shyam Chand, Diana Sahy, Daniel J. Condon, Stephen R. Noble, Tõnu Martma, Terje Thorsnes, Simone Sauer, Harald Brunstad

**Affiliations:** 1 Marine Geology, Geological Survey of Norway, Postal box 6315 Sluppen, 7491 Trondheim, Norway; 2 CAGE—Centre for Arctic Gas Hydrate, Environment and Climate, Department of Geology, UiT the Arctic University of Norway, 9037 Tromsø, Norway; 3 Institute of Geology, Tallinn University of Technology, 19086 Tallinn, Estonia; 4 Department of Geology, University of Tartu, Ravila 14A, 50411 Tartu, Estonia; 5 British Geological Survey, Keyworth, Nottingham NG12 5GG, UK; 6 Lundin Norway AS, 1366 Lysaker, Norway

## Abstract

Gas hydrates stored on continental shelves are susceptible to dissociation triggered by environmental changes. Knowledge of the timescales of gas hydrate dissociation and subsequent methane release are critical in understanding the impact of marine gas hydrates on the ocean–atmosphere system. Here we report a methane efflux chronology from five sites, at depths of 220–400 m, in the southwest Barents and Norwegian seas where grounded ice sheets led to thickening of the gas hydrate stability zone during the last glaciation. The onset of methane release was coincident with deglaciation-induced pressure release and thinning of the hydrate stability zone. Methane efflux continued for 7–10 kyr, tracking hydrate stability changes controlled by relative sea-level rise, bottom water warming and fluid pathway evolution in response to changing stress fields. The protracted nature of seafloor methane emissions probably attenuated the impact of hydrate dissociation on the climate system.

## Abstract (web summary)

Understanding the timescales of gas hydrate dissociation and methane release are critical to gauge the potential climate impact. Here, the authors report a methane efflux chronology from five sites in Barents and Norwegian seas and show methane release coincident with the release of ice sheet-induced pressure.

## Graphical abstract

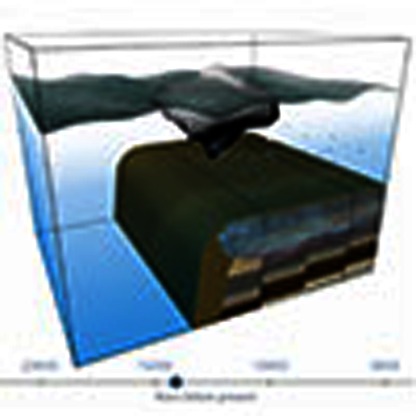

Large quantities of methane exist as free gas, dissolved in pore fluids and solid gas hydrates within marine sediments^1^. Gas hydrates are found in a variety of geological settings^2^ but are most common on continental margins where the combination of appropriate pressure–temperature conditions and the presence of organic-rich sediments are conducive to abundant methane generation. The stability of the marine gas hydrate reservoir is primarily affected by changes in temperature and hydrostatic pressure. Consequently, changes in the thickness of the gas hydrate stability zone (GHSZ) are likely to occur along glaciated continental margins where pressure regimes are governed by alternating glacial loading and unloading cycles, and gas hydrate dissociation may be amplified by the influx of warmer waters following ice retreat^2^,^3^,^4^,^5^. If bottom water warming and/or hydrostatic pressure decrease is large enough, gas hydrate dissociation and release of a significant amount of methane are possible^4^,^6^,^7^,^8^,^9^,^10^. The transfer of this methane to the water column has the potential to amplify ocean acidification and de-oxygenation and, possibly, atmospheric greenhouse gas concentrations^11^,^12^. The rates and timescales over which gas hydrates dissociate control the impact of hydrate-derived methane on the global climate system.

Methane released by the dissociation of marine gas hydrates has been inferred to be the cause of a number of negative δ^13^C excursions in the geological record^6^,^7^. Large magnitude gas hydrate dissociation events are also proposed to have occurred in the more recent past, as evidenced by major fluid flow structures in sedimentary basins, such as pockmarks^4^,^13^. However, the role of marine gas hydrate releases in influencing atmospheric methane concentrations during the Quaternary remains uncertain. For example, the radiocarbon and hydrogen isotopic composition of methane from Greenland ice cores suggests that atmospheric methane rise at 11.6 and 38 ka can be predominantly accounted for through wetland emissions^14^,^15^. Although it appears that Quaternary marine gas hydrate dissociation events did not have a significant impact on the climate system, recent estimates of the gas hydrate reservoirs stored beneath the grounded Antarctic ice sheet suggest that methane release resulting from accelerated ice wastage has the potential to act as a positive feedback on future climate warming^16^.

Methane is consumed at a variety of stages during transport through the sediment and overlying water column. On a global scale, >80% of methane migrating within the sedimentary package can be microbially consumed at the sedimentary sulphate–methane transition zone by the anaerobic oxidation of methane (AOM)^11^,^17^. However, where gas supply is high and flow through the sediment is advective, the capacity of this benthic microbial filter can be outstripped, resulting in release of methane into the overlying water column^18^. A proportion (up to 100%) of methane within the water column is consumed via dissolution of free gas bubbles and methanotrophic microbial activity^19^,^20^ before it reaches the ocean surface and escapes into the atmosphere. Consequently, the short residence time of methane in the oceans and atmosphere (∼10 years), combined with the processes that control consumption in the sediment and water column, requires a rapid (decadal) release and transfer of large volumes of methane to achieve a significant impact on ocean/atmosphere chemistry.

The processes that influence gas hydrate stability in continental margins act on timescales ranging from near instantaneous (for example, slope failure) to 10^2^–10^3^ years (ice sheet collapse), whereas significant sea-level and bottom water temperature variations operate over 10^3^ years^21^,^22^. The transfer of methane from the seabed into the water column is typically detected by water column acoustic gas flares, with observations restricted to timescales of less than a few decades^20^,^23^. However, assessments of the timescales over which gas hydrate systems respond to the processes that drive changes in pressure and temperature in the subsurface are limited. Studies from GHSZ pinch outs indicate rapid response to temperature change in the immediate subsurface over a few decades^8^,^22^. Modelling results show that heat transfer and temperature re-equilibration of ∼100 m of marine sediments would take 10^2^–10^3^ years^8^,^24^, and that gas propagated from such depth may take an additional thousand years to vent at the seafloor^24^. However, empirical constraints on methane efflux triggered by hydrate dissociation resulting from glacial unloading of a continental margin, both past and present are, as yet, lacking.

Direct information on the timing of past methane release events can be obtained through U-Th dating of methane-derived authigenic carbonates^25^,^26^, which form at the sedimentary sulphate–methane transition zone, due to elevated carbonate alkalinity resulting from AOM in areas experiencing relatively intense methane fluxes over 10^2^–10^3^ years^25^. To date, U-Th dating of methane-derived carbonates has mostly focused on samples from mid-latitudes and was used to assess the periodicity of fluid migration along fault systems^27^,^28^ and the effects of long-term changes in sea-level and bottom water temperature on methane efflux and/or gas hydrate dissociation^26^,^29^,^30^. Conversely, U-Th dating of carbonates from methane seeps off Svalbard indicates that the stability of gas hydrates at the updip of the GHSZ at high-latitude sites is sensitive to seasonal bottom water temperature fluctuations^21^.

The shelf areas of the Norwegian and Barents Seas offer ideal settings in which to assess the processes governing gas hydrate stability on glaciated continental margins with grounded ice and hence can serve as a palaeo-analogue of the Antarctic gas hydrate reservoir^16^. Here we report on the timing of methane release in the Barents and Norwegian Seas, where a drop in seabed pressure associated with the retreat of the grounded Scandinavian Ice Sheet (SIS) has been inferred as the driving force behind the dissociation of a significant amount of gas hydrates^3^,^13^,^31^ (Fig. 1). We use U-Th geochronology, stable carbon and oxygen isotope analyses and petrographic observations on authigenic carbonate crusts associated with methane seeps to provide constraints on the timescales of past methane fluxes from five locations for that region. Our new U-Th data set, combined with model estimates of the amount of methane potentially stored as gas hydrates before and after the collapse of the SIS offers an opportunity to study the timescales of methane efflux across the seafloor in response to the environmental changes associated with the last deglaciation.

## Results

### Studied areas

The southwest Barents and Norwegian Sea are active provinces for oil and gas exploration, with extensively documented, focused, hydrocarbon-rich fluid migration through regional fault networks^3^,^32^,^33^. Mega-scale glacial lineations (for example, in the Ingøydjupet depression) indicate that the shelf areas of those seas were covered by the grounded SIS during the last glaciation^34^,^35^,^36^. The pressure exerted by grounded ice on the underlying sediments was associated with a ∼500 m thickening of the GHSZ^3^,^37^ and reactivation of faults and fractures as fluid pathways. Age constraints on grounded ice wedges indicate that ice persisted on the southwest Barents Sea shelf at least until 17 ka^38^,^39^, and that ice streams extended to the edge of the shelf at least twice^34^,^36^. In this area the seafloor is marked by ubiquitous pockmarks, circular depressions resulting from the expulsion of fine-grained sediments in response to fluid seepage^3^,^13^,^31^,^40^,^41^. Although some pockmarks coincide with observed water-column gas flares, indicating active seepage, most are inactive and constitute records of widespread fluid escape at the seafloor related to abrupt gas hydrate dissociation after the last deglaciation^3^,^13^,^31^. The cross-cutting relationships between pockmarks and surrounding sediments, coupled with radiocarbon dating, indicate that pockmark formation post-dated the deposition of glaciomarine sediments at ∼15 ka^31^.

**Figure 1:**
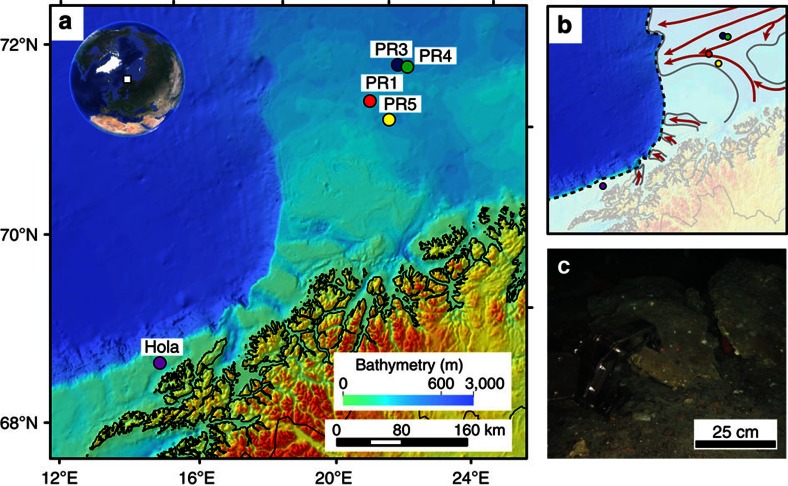
Bathymetric map of the study area and remotely operated underwater vehicle (ROV) recovery of authigenic carbonate. (**a**) The five carbonate sampling sites (PR1, PR3, PR4, PR5 and Hola) are shown as circles. (**b**) The white shaded area represents the extent of the Scandinavian Ice-sheet during the LGM^51^ with main ice streams depicted by red arrows^35^,^39^. (**c**) Carbonate crust sampled at the seafloor.

An autonomous underwater vehicle was used to map the seabed at high spatial resolution (<10 cm) in some pockmark areas and areas of known seepage^3^,^42^, and revealed the presence of numerous carbonate crusts. Guided by the autonomous underwater vehicle mapping results, carbonate crusts were sampled by a remotely operated underwater vehicle from four sites in the southwest Barents Sea at depths ranging between 320 and 400 m (PR1, PR3, PR4 and PR5; Fig. 1 and Table 1). Additional samples were collected from the Hola site (depth 218 m) off the Vesterålen islands in the Norwegian Sea. Active gas seepage, inferred from gas flares in the water column, occurs at all sites except at PR5.

### Carbonate crust characterization

**Figure 2:**
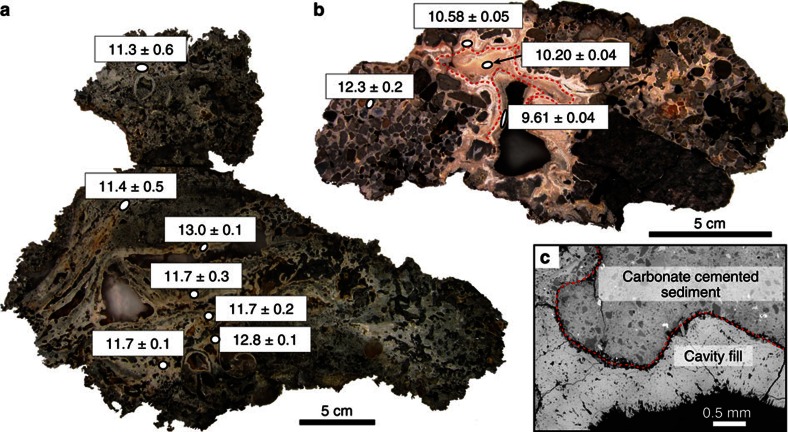
Authigenic carbonate crusts. Detailed U-Th ages (in ka±2*σ*) on representative cross-sections of (**a**) sample P1210002 and (**b**) sample P1210017. (**c**) Scanning electron microscope image showing early-stage cements containing detrital material and detritus-free layer of radial fibrous aragonite cavity fill.

The typical thickness of studied crust samples varies between 10 and 30 cm (Fig. 2). Authigenic carbonates occur as impure early-generation cements, lithifying mud to gravel-size detrital glacial/glaciomarine sediments and as pure detritus-free later-generation botryoidal-layered fillings of centimetre-scale cavities formed within the early-generation carbonate-cemented sediments (Fig. 2b,c). Such cavities are interpreted as conduits generated by a focused fluid flow, which washed out weakly cemented sediments. Microcrystalline and radial fibrous aragonite cements are the dominant authigenic carbonate phases, but minor micritic Mg-calcite (13–15% mol MgCO_3_) is also present (Supplementary Fig. 1 and Supplementary Table 1). Both aragonite and high Mg-calcite cements contain disseminated framboidal pyrite indicative of active microbial sulfate reduction during carbonate precipitation^43^. The dominance of aragonite among diagenetic carbonate phases probably indicates precipitation under methane fluxes capable of sustaining high rates of AOM activity in sediments just beneath the seafloor^44^.

**Figure 3:**
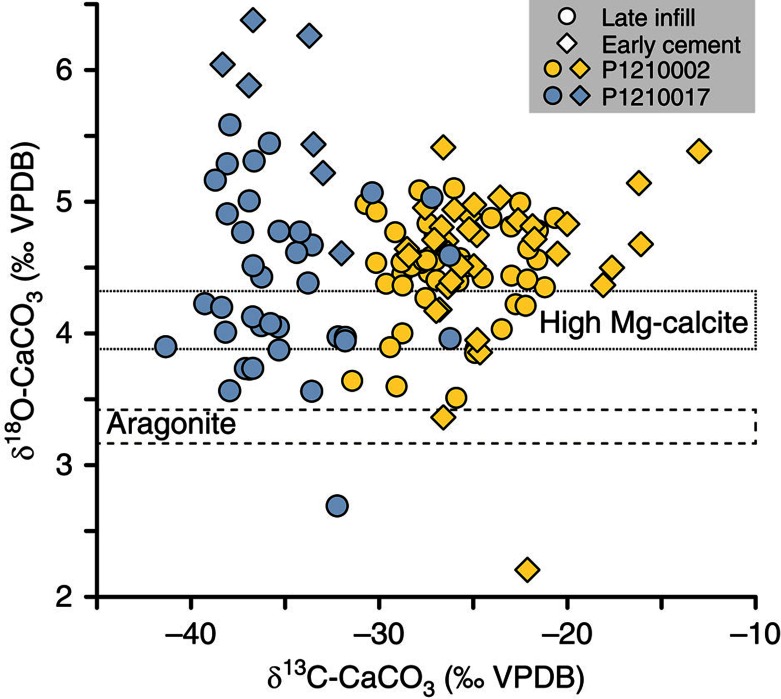
Stable C and O isotopes. Isotope compositions of early generation cements and late generation cavity infills of samples hand-drilled from two representative carbonate crusts. The boxes deliniated by dashed lines indicate the δ^18^O range of carbonate (aragonite^47^ and Mg-calcite with 13–15% mol MgCO_3_ (refs 48, 49)) precipitating in equilibrium with present-day bottom water (temperature of 2–3 °C and seawater δ^18^O of 0‰ VSMOW).

Carbon and oxygen isotope compositions of the sampled carbonate crusts were measured on powders hand-drilled on a millimetre scale, to spatially resolve distinctive carbonate generations. Carbonate δ^13^C values range from −43.1 to −13.0‰ Vienna Pee Dee Belemnite (VPDB) (Fig. 3 and Table 1, average −32.3±4.3‰ VPDB), indicating that microbial oxidation of methane-rich fluids is the dominant source of carbon. The absence of very ^13^C-depleted (δ^13^C <−45‰ VPDB) carbonates in the studied crusts further suggests that the principal source of methane is thermogenic, which is typically less ^13^C depleted than microbial methane^45^. A predominantly thermogenic origin is also consistent with the stable isotope composition of methane gas currently seeping from the seabed (δ^13^C-CH_4_ of −48‰ and δD-CH_4_ of −186‰ Vienna Standard Mean Ocean Water (VSMOW); Supplementary Fig. 2).

The δ^18^O values of the authigenic carbonates provide information about the temperature and oxygen isotope composition of the ambient porewater in which carbonates precipitate. The oxygen isotope composition of carbonates precipitating in equilibrium with the bottom water after deglaciation, that is, at 15 ka (δ^18^O=1‰ VSMOW^46^ and 1 °C) would have a δ^18^O value of 4.7‰ VPDB for aragonite^47^ and 5.6‰ VPDB for Mg-calcite with 15% mol MgCO_3_ (refs 48, 49). The high δ^18^O values (that is, up to 6.4‰ VPDB; Fig. 3) measured in the early-generation Mg-calcite-cemented sediments cannot be explained by precipitation of Mg-calcite in equilibrium with the bottom waters during the early deglaciation period and probably indicate the influence of ^18^O-rich water potentially released during gas hydrate dissociation^50^.

### U-Th geochronology

**Figure 4:**
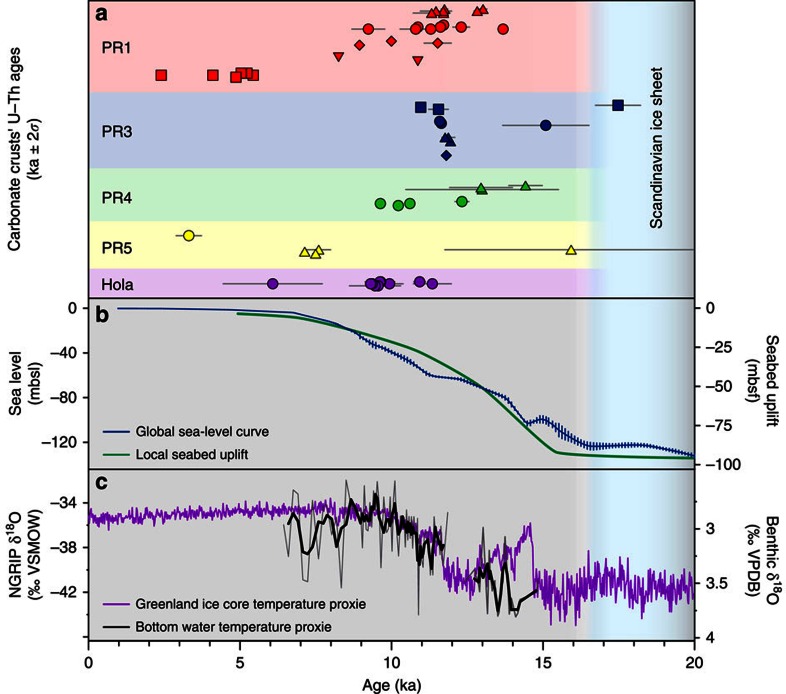
U-Th carbonate crust dating results compared with paleoclimate and sea-level records since the LGM. (**a**) U-Th ages (error bars are 2*σ*) on 14 studied carbonate crusts (up to 11 ages per crust) grouped by site (see Fig. 1 for locations). (**b**) Global sea-level curve (error bars are 1*σ* (ref. 61)) and seabed uplift curve (extracted from ref. 57). (**c**) Stable oxygen isotope records of ice core, North Greenland Ice Core Project (NGRIP)^75^ and of benthic foraminifera from a sediment core from the southwest Barents Sea^76^. The blue vertical shaded area depicts the period when grounded ice-sheet prevailed across the study area^39^.

A total of 14 methane-derived authigenic carbonate crusts from the five sites were selected for U-Th dating; the sampling targeted both early-stage carbonate cements and late-stage cavity fills (Supplementary Data 1). The latter are characterized by low detrital content ([^230^Th/^232^Th]_AR_ ranging between 5 and 427) and give dates with a precision on the order of ±0.5–1% (2*σ*). Early-stage cements prove to be more difficult to date effectively, owing to the presence of a high proportion of detrital material (Fig. 2c), with uncertainties magnified by the impact of the corrections required to account for the presence of initial detrital Th (Fig. 2b and Supplementary Data 2). We obtain 59 reliable dates (defined as [^230^Th/^232^Th]_AR_ >2, see Fig. 4 and Supplementary Note), mostly from late-stage cavity fills, which show that 12 of the analysed crusts formed from 17 to 7 ka. Younger dates, between 5 and 2 ka, are obtained from two crusts, one each from the PR1 and PR5 sites (Fig. 4). Owing to the preponderance of late-stage aragonite cavity fills, the U-Th data set provides minimum age estimates for the onset of emissions, which is recorded by the less well-dated early-stage cements. Both the older (17–7 ka) and younger (5–2 ka) crusts span several 100–5,000 years (Fig. 4) and constrain the minimum duration and absolute timing of methane efflux at each site. Data from multiple subsamples collected from the same crust are consistent with the observed textural relationships (that is, cavity fills are younger than early-stage cements; Fig. 2b).

### Gas hydrate stability modelling

**Figure 5:**
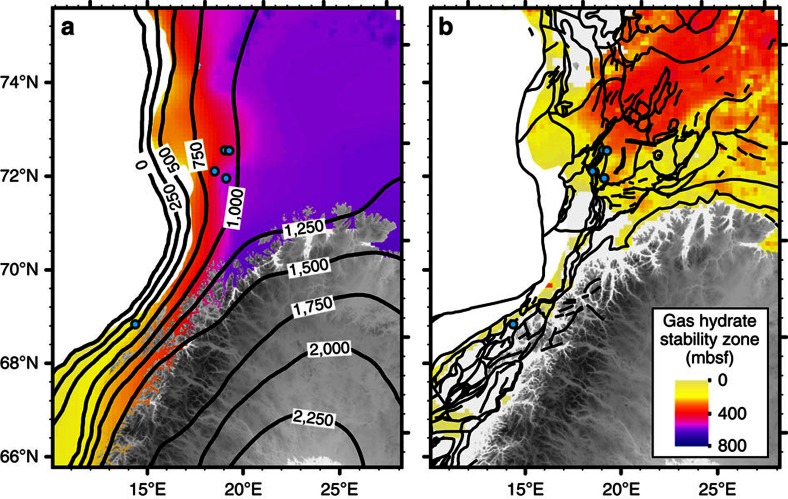
Steady-state modelled thickness of the GHSZ. Thickness of GHSZ during the LGM (**a**) and at present-day conditions (**b**). Black contours denote (**a**) the ice-sheet thickness in metres during the LGM^51^ and (**b**) broad scale faulting system^77^. The average value of the geothermal gradient used is 31 °C km^−1^ (ref. 52). It is noteworthy that the abyssal plain has not been taken into account in our model.

Gas hydrate stability was modelled at steady state based on the presence of an ∼1,100-m-thick grounded ice sheet^51^, the regional average geothermal gradient of 31 °C  km^−1^ (ref. 52) and a gas composed of 96% methane, 3% ethane and 1% propane. Such a gas composition is consistent with the base of the GHSZ corresponding to the observed bottom-simulating seismic reflectors in the Barents Sea^37^,^53^. The model estimates the thickness of the GHSZ at up to 600 m below the seafloor during the Last Glacial Maximum (LGM), thinning gradually towards the shelf edge due to decreasing ice load (Fig. 5a). Following the retreat of grounded ice, which triggered isostatic rebound, sea-level rise and bottom water warming, the GHSZ thinned to *ca*. 100 m in most of the southwest Barents Sea, including the study area. A thicker GHSZ of up to 400 m persists in the deeper, central part of the southwest Barents Sea (Fig. 5b). Based on the volume change of gas hydrates between the LGM (Fig. 5a) and the present day (Fig. 5b), we estimate that 5–43 Gt of methane was released in the studied area (4 × 10^5^ km^2^).

## Discussion

The clustering of U-Th dates indicates that the main crust-forming methane flux episode took place between 17 and 7 ka (Fig. 4), which is consistent with the estimated timing of pockmark development in the Barents Sea^31^ and North Sea^54^, suggesting that fluid release activity was controlled by a regional process. Although the onset of crust growth is less well constrained, the oldest U-Th age (17.5±0.7 ka) coincides with the deglaciation of the southwest Barents Sea (∼18–16 ka) (Fig. 4), which is constrained by ^14^C dating of foraminifera associated with glaciomarine sediments^39^. The U-Th ages and the presence of isotopically heavy oxygen (>5‰ VPDB) support a model in which methane is discharged from gas hydrate dissociation, triggered by pressure changes on the continental shelf due to collapse and retreat of the SIS^3^,^31^. The U-Th data further indicate that substantial methane efflux continued along the ice-free northern Norwegian margin for another *ca*. 10 kyr. Although the carbonate crust U-Th data are limited in coverage, the clustering of U-Th dates (Fig. 4) does suggest that methane efflux may have been pulsed. U-Th ages of distinct layers within cavity infills provide evidence for sustained methane fluid flow through individual conduits over 900 years (Fig. 2b). Such focused fluid flow events may be analogous to the observed active water column acoustic gas flares and as such could provide a means to extrapolate observed fluxes.

**Figure 6:**
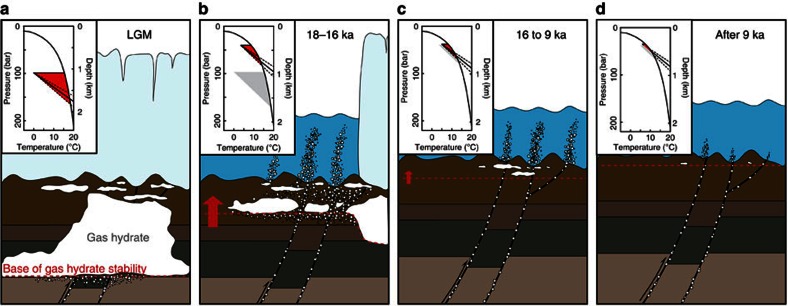
Schematic sketch illustrating different snapshots of gas hydrate stability at steady state and fluid flow dynamics through time in the southwest Barents Sea shelf. (**a**) During the LGM, gas hydrate stability shown with the red area in the top-left corner was extending up to 600 m below the seabed. (**b**) Methane migrates through fractures and porous media as a result of gas hydrate dissociation triggered by grounded ice sheet retreat 18–16 ka. (**c**) Gas hydrate dissociation continues during the isostatic rebound and bottom water warming from ∼16 to ∼9 ka. (**d**) After ∼9 ka to present, gas plumes occur locally connected to open deep-seated faults. The average geothermal gradient and associated 2*σ* uncertainties (31±6 °C km^−1^ (ref. 52)) are shown by solid and dashed lines, respectively, at the base of gas hydrate stability fields (red areas). The red arrow depicts relative change of the base of the GHSZ (red dashed line). Temperature and pressure constraints used for assessing change in GHSZ are in Supplementary Table 3.

The integrated geochemistry and geochronology of methane-derived authigenic carbonates, coupled with gas hydrate modelling, provides a conceptual and temporal framework for methane seepage in the southwest Barents Sea. During the LGM, the SIS covered most of Northern Europe, including the Norwegian continental shelf and the entire Barents Sea^55^,^56^. Glacial loading by about 1,100 m of grounded ice^51^ created a 150- to 200-m isostatic depression of the lithosphere^57^ and the resulting increase of hydrostatic pressure in the underlying sediments extended the GHSZ to up to 600 m below the seafloor throughout the Barents Sea and the Norwegian shelf (Figs 5a and 6). Ice loading probably reactivated widespread basement-penetrating fault systems enhancing the migration of gas originating in Triassic and Jurassic source rocks and hydrocarbon reservoirs^58^. The accumulation of thermogenic gas in the upper part of the sediment column, corresponding to the GHSZ, would have enabled widespread gas hydrate formation beneath the ice cap during the LGM (Fig. 6a).

The retreat of the SIS from the shelf margin began at 19 ka and comprised several episodes of ice retreat and advance with grounding zone wedge formation^35^,^39^. The western Barents Sea, including our study area, was deglaciated over a 2-kyr period, with ice-free conditions reached at ∼16 ka^39^. The pressure drop associated with ice sheet unloading led to thinning of the GHSZ by as much as 400 m, resulting in gas hydrate dissociation and thereby increased pore pressure, triggering methane advection (Fig. 6b). Assuming a Darcy linear flow model, the dissipation of pore pressure from the seafloor to the base of the LGM GHSZ (600 m below seafloor) during glacial retreat is expected to have taken between 0.005 and 4.6 kyr, to reach the steady state (permeability of 0.04 mD to 1.51 D^59^ and water viscosity of 2.04 × 10^−3^ Pȧs). In addition, the rate of gas hydrate dissociation, position within the sedimentary column and the fluid flow velocity would potentially affect the time over which the fluids reach the seafloor. As carbonate crusts are forming at fluid flow velocities between 20 and 60 cm per year^60^, the methane migration at such velocities from the base of the LGM GHSZ (600 m) to the seafloor would take between 1 and 3 kyr.

Following the SIS collapse, isostatic rebound caused a *ca*. 90 m uplift of the seafloor between 16 and 6 ka (Fig. 4), and at the same time sea-level rose *ca*. 120 m (ref. 61), resulting in a minor net increase in hydrostatic pressure. Changes in stress over this time interval associated with glacial unloading and isostatic rebound resulted in reactivation of pre-existing faults in certain orientations relative to the evolving stress field^62^ and would have facilitated fluid migration from shallow gas reservoirs generated by gas hydrate dissociation and/or from deeper petroleum systems. Gas hydrate dissociation was further promoted by the inflow of warm Atlantic water into the Barents Sea shelf after deglaciation, as bottom water temperatures rose from 4 °C at ∼15 ka to 6 °C at ∼10 ka with 1–2 °C fluctuations on 100-year timescales (Fig. 4), remaining relatively stable during the Holocene. The heat transfer would take several hundred years to propagate through the sediments and reach the base of the GHSZ^24^. Consequently, post-glacial gas hydrate destabilization possibly continued until ∼9–7 ka under the combined influence of seabed uplift and temperature increase (Fig. 6c). At present, gas seepage as evidenced by flares in the water column in the Barents Sea is mainly observed above deep-seated faults (Fig. 6d) with methane sourced from petroleum reservoirs.

We estimate that 10 × 10^3^ – 88 × 10^3^ Tg of methane was stored as gas hydrate in the southwest Barents Sea shelf during the LGM, which is equivalent to 0.6–4.9% of the current total oceanic gas hydrate reservoir^63^. As our study area only represents *ca*. 30% of the glaciated Barents Sea shelf and *ca*. 5% of all glaciated shelves with grounded ice sheets (that is, Norwegian margin, Antarctica and Greenland), it is likely that significant amounts of methane were stored in such shelf settings during the LGM. However, the impact of oceanic gas hydrate dissociation on the climate system is limited by the atmospheric transfer rate of methane, which is a function of timescales of dissociation and release rates at the seafloor, the efficiency of methane oxidizers and bubble dissolution in the water column, and the thickness of the water column. Modelling estimates show that under normal oceanographic conditions, methane released at water depths >200 m would be almost entirely (>80%) consumed during transit towards the sea surface^20^, and that a catastrophic bubble release is required, to drive transport of methane from deeper waters (>100 m) to the atmosphere^20^. As water depth in the investigated areas of the Norwegian and Barents Sea was on the order of 200–400 m after ice sheet collapse, the resulting impact on atmospheric methane concentrations was probably muted. Furthermore, methane release at the sediment–water interface was also probably modulated by the availability of excess methane and fluid pathways to the seafloor, with slower transit through the sediment column increasing the magnitude of microbial methane consumption within the sedimentary sulphate methane transition zone. Assuming that 90% of the methane released by gas hydrate dissociation is consumed by AOM^11^, and that 1–5% of it would pass through the water column and reach the atmosphere, the mean integrated flux to the atmosphere in the southwest Barents Sea over 10 kyr would be about 0.0005–0.0172 Tg·per year (minimum and maximum estimates) of methane, which is relatively small when compared with the ∼200 Tg·per year methane flux from all natural sources^64^. Such a scenario of protracted, relatively low-intensity methane efflux in response to abrupt environmental changes has rarely been evaluated by studies that postulate linkages between gas hydrate dissociation and transfer of methane to the marine realm/atmosphere. However, on shallow continental shelf regions (that is, <150–200 m water depth) that have also experienced glacial–interglacial cycles, it is plausible that a higher proportion of the methane released by gas hydrate dissociation may have passed through the water column and reached the atmosphere.

The main episode of carbonate crust formation in the Barents Sea after the collapse of the SIS broadly overlaps with elevated atmospheric methane concentrations as recorded by ice cores from Greenland and Antarctica^65^. The potential importance of gas hydrate destabilization due to warming of upper oceanic water masses as a cause for elevated atmospheric methane has been debated in several publications^4^,^10^. The hydrogen isotope and ^14^C characteristics of methane trapped in ice cores suggest insignificant emissions from marine gas hydrates during times of high atmospheric methane after deglaciation and stability of gas hydrates^14^,^66^. However, the general inference of gas hydrate stability within continental margin sediments through glacial–interglacial cycles is not globally applicable, as it is dependent on local changes in temperature and hydrostatic pressure. This is particularly important in case of glaciated continental margins where gas hydrate destabilization was triggered by local reduction in pressure effects of collapsing grounded ice sheets. Reliable assessment of the influence of hydrate-released methane from glaciated margins on the climate system after the LGM requires global quantification of methane storage, release and consumption budgets, constraints of timescales of hydrate dissociation, and temporal and volume estimates of the dynamics of ice sheets. Such global data are not currently available. Abrupt, globally synchronous methane release over a timescale of 10^2^ years from deglaciated shelf areas with grounded ice appears unlikely given the protracted nature of hydrate-derived methane efflux after the LGM, as our findings from the southwest Barents Sea indicate, as well as the asynchronous deglaciation of different shelf areas.

The analysis of methane-derived authigenic carbonate through the integration of U-Th geochronology and geochemical proxies, combined with gas hydrate modelling provides a means for evaluating past methane release from glaciated continental margins where gas hydrate dynamics are governed by glacial–interglacial cycles. Modelling results demonstrate that gas hydrate accumulation beneath grounded ice-sheets on the Norwegian margin, in a setting analogous to the present day Antarctic shelf^16^, generates potentially significant methane reservoirs the stability of which is sensitive to environmental changes affecting local pressure and temperature regimes. Although significant amounts of methane could have been released at the seafloor and transferred to the water column, U-Th geochronology suggests methane release over a *ca*. 10 kyr interval implying that flux rates were modulated by the second-order processes controlling both the rate of dissociation (isostatic rebound and bottom water warming), fluid transport (changes in stress fields leading to fault reactivation under the control of isostatic rebound) and consumption (via efficiency of microbial oxidation). Overall, the protracted nature of methane release on the Norwegian margin and its minimal impact on atmospheric methane concentrations highlight the complexity of the gas hydrate system and the importance of mechanisms mediating gas hydrate dissociation and fluid advection in response to abrupt climatic change.

## Methods

### Mineralogy

Petrographical characterization of carbonates was achieved by a combination of optical and scanning electron microscopy techniques. Mineralogy was determined at the Geological Survey of Norway by X-ray diffraction (Philips X'pert apparatus, Cu K_α_ radiation in 2–70° 2*θ* range) on bulk carbonate powder. Identification of the main carbonate phases was done using EVA software (Supplementary Table 1). The *d*_104_ displacement was used to estimate the MgCO_3_ mol% in calcite^67^.

### Stable isotope compositions

Stable carbon and oxygen isotopes of hand-drilled carbonate were analysed with a GasBench II preparation line connected to a Thermo Scientific Delta V Advantage IRMS (Thermo Fisher Scientific). Reproducibility is better than ±0.2‰ for both δ^13^C and δ^18^O. The stable carbon and hydrogen isotopes of methane (Supplementary Fig. 2) were analysed by a Delta plus XP IRMS (Thermo Fisher Scientific) with analytical reproducibility better than ±1‰ for δ^13^C values and better than ±10‰ for δD values, respectively. Stable isotope compositions are reported in conventional delta (*δ*) units relative to the VPDB reference for carbon and oxygen, and relative to the VSMOW reference for hydrogen isotopes.

### U-Th dating

U-Th dating was carried out on 76 microdrilled carbonate samples weighing between 2–80 mg (Supplementary Data 1 and Supplementary Fig. 3). A further seven unconsolidated sediment samples (three to six replicates from each, weighing 30–130 mg) were also analysed, to constrain the impact of incorporated detrital material on the U-Th systematics of the carbonate crusts. Analytical protocols for the separation and purification of U and Th from carbonate crust and detritus samples are similar to those of Douarin *et al*.^68^, with modifications aimed at ensuring the following: (i) complete dissolution of the detrital material incorporated in the crusts and (ii) oxidation of organic material liable to produce isobaric interferences during measurements of Th isotope ratios^69^. All evaporation steps took place in a closed EvapoClean device, to minimize cross-contamination and reduce fall-in blanks. Samples were leached in ∼8 M HNO_3_ for 1 h and centrifuged to separate soluble carbonate-rich fractions from less-soluble detrital material. Detritus fractions were dissolved over 1–7 days in a mixture of HClO_4_:HF:HNO_3_ (1:2:2.5), using ∼50 μl HF per estimated mg of material, dried down, re-dissolved in 8 M HCl and dried down again, to ensure the conversion of residual fluorides to chlorides. Detritus fractions were then dissolved in 8 M HNO_3_, recombined with their respective carbonate fractions, spiked with a mixed ^229^Th-^236^U tracer, left to equilibrate overnight and dried down. All samples went through two overnight oxidation steps in a mixture of 2 ml 16 M HNO_3_ and 0.2 ml 30% H_2_O_2_ followed by evaporation to dryness. Pre-concentration of U and Th through Fe co-precipitation and initial separation on 0.6 ml columns using AG-1 × 8 anion exchange resin were done following the procedure of Edwards *et al*.^70^. Th fractions were further purified using a second pass through AG-1 × 8 resin and were filtered using 0.22-μm pore-size syringe filters to remove resin particles. Both U and Th fractions were oxidized twice in 2 ml 16 M HNO_3_ and 0.2 ml 30% H_2_O_2_, and dissolved in 1 ml 0.1 M HCl and 0.035 M HF. Before mass spectrometry analyses, all samples were filtered to remove particles originating from the perfluoroalkoxy alkane (FEP) beakers used for sample preparation.

Isotope ratio measurements were made on a Thermo Neptune Plus multi-collector ICP-MS, with samples introduced via an Aridus II desolvating nebulizer using an ESI PFA nebulizer tip with an ∼50 μl min^−1^ uptake rate. Typical operating conditions included the addition of 4–8 ml min^−1^ high-purity N_2_ to the sample-carrying Ar stream, to minimize U and Th oxide formation in the plasma.

U measurements were made using a normal sample cone and X-skimmer cone, and a static multicollector data collection protocol with ^234^U measured on an axial secondary electron multiplier, and ^233^U, ^235^U, ^236^U and ^238^U measured on Faraday cups equipped with 10^11^ Ω resistors. Blocks of five samples were bracketed by analyses of CRM 112a U and CRM 112a U+IRMM 3636 spike. Exponential mass bias corrections were based on the measured values of the ^233^U/^236^U ratio of the IRMM 3636 spike normalized to a value of 1.01906. Secondary electron multiplier/Faraday gain corrections were based on the ^234^U/^235^U ratio of bracketing unspiked CRM 112a analyses. Hydride formation and tailing were monitored at the beginning of each analytical session, with measurements made at mass 237 and 239, while aspirating an unspiked CRM 112a solution, and were corrected during offline data reduction.

Owing to the young age of the samples, Th measurements required better sensitivity and were predominantly made using a Jet sample cone and X-skimmer cone. ^230^Th was measured on the axial secondary electron multiplier, with ^229^Th and ^232^Th measured on Faraday cups equipped with 10^11^ Ω resistors. Blocks of ten samples were bracketed by blocks of five analyses of an in-house ^229^Th-^230^Th-^232^Th reference solution calibrated against CRM 112a, which was used to monitor mass bias and secondary electron multiplier/Faraday gain.

U-Th age calculations were performed in Isoplot v. 3.75 (ref. 71). The impact of measured detritus ^232^Th/^238^U activity ratio variability on the geological interpretation of U-Th dates within the scope of this study was found to be negligible for samples with (^230^U/^232^Th) activity ratios above ∼2 (see Supplementary Note). Corrected U-Th dates from samples with (^230^Th/^232^Th)<2 (*n*=16) were either negative or statistically equivalent to zero (at the 2*σ* level) and were consequently excluded from the data set (Supplementary Data 1 and 2). The remaining analyses were corrected using a calculated average composition of carbonate-free detritus samples (Option 3, Supplementary Table 2).

### Gas hydrate modelling

The gas hydrate stability modelling was carried out assuming steady state using CSMHYD software^72^. Consequently, this approach illustrates only changes in gas hydrate stability independent of the transient behaviour of the system. We used the bathymetry grids of the southwest Barents Sea shelf (https://www.ngdc.noaa.gov/mgg/global/global.html) and World Ocean Database 2005 bottom water temperature (http://www.nodc.noaa.gov/OC5/indprod.html) from NOAA and a feeding thermogenic gas composition of 96% methane, 3% ethane and 1% propane^37^. As the underlying overturned sediments have varying consolidation with a range of different thermal conductivity and hence heat flow values^59^, the geothermal gradient in Barents Sea can vary from 22.8 to 69.3 °C km^−1^, but is mostly confined around 31±6 °C km^−1^ (2*σ*)^52^. For the Last Glacial Maximum (LGM), ice thickness models predicted for the Barents Sea varies in coverage and thicknesses, ranging from 750 to 2,000 m in the study area^51^,^56^,^73^. For predicting the gas hydrate stability during the LGM model, we used the data from ref. 56, which predicts an ice-sheet thickness of ∼1,100 m for the southwest Barents Sea falling within the most accepted range and we applied a bottom ice temperature of −1.5 °C.

The model of gas hydrate stability was used to estimate the volume of the GHSZ (12.7 × 10^4^ km^3^) in the southwest Barents Sea shelf during the LGM. Considering a sediment porosity^58^ of 0.1–0.3 conservatively filled by 1–2% of gas hydrate in which methane occupies 70–100% of the cages^74^, the weight of methane stored in gas hydrate would range between 10 and 88 Gt, calculated with a volumetric ratio between methane and gas hydrate of 164 and a methane density of 0.70805, kg m^−3^ at standard conditions for temperature and pressure (0 °C and 1 bar).

## Additional information

**How to cite this article**: Crémière, A. *et al*. Timescales of methane seepage on the Norwegian margin following collapse of the Scandinavian Ice Sheet. *Nat. Commun.* 7:11509 doi: 10.1038/ncomms11509 (2016).

## Supplementary Material

Supplementary Information Supplementary Figures 1-3, Supplementary Tables 1-3, Supplementary Note 1 and Supplementary References.

Supplementary Data 1 U-Th isotopic data

Supplementary Data 2 U-Th detritus-corrected age

## Figures and Tables

**Table 1:** U-Th isochron ages, stable carbon and oxygen isotopic composition, coordinate and water depth for carbonate samples.

**Area**	**Sample**	**U-Th age (ka**±**2*****σ*****)**			**δ**^**13**^**C**	**δ**^**18**^**O**	**Latitude N**	**Longitude W**	**Water depth (m)**
		**Min.**	**Max.**	***n***	**(‰ VPDB)**	**(‰ VPDB)**			
*PR1*									
	P1201001	9.2±0.5	13.7±0.1	8	−30.5 to −18.1	4.0 to 5.3	72° 09' 28.5"	19° 43' 38.5"	319
	P1210002	11.3±0.6	13.0±0.1	7	−31.4 to −13.0	3.4 to 5.4	72° 09' 28.1"	19° 43' 37.7"	320
	P1210004	8.9±0.1	11.5±0.5	3	−35.5 to −23.0	2.8 to 5.9	72° 09' 27.8"	19° 43' 38.0"	320
	P1210006	2.38±0.03	5.4±0.1	6	−43.1 to −33.4	3.0 to 4.2	72° 09' 28.0"	19° 43' 38.7"	320
	P1210007	8.2±0.1	10.9±0.1	2	−33.8 to −19.5	3.7 to 5.4	72° 09' 27.8"	19° 43' 37.9"	320
*PR3*									
	P1210010	11.6±0.1	15.1±1.4	3	−32.6 to −27.6	4.4 to 5.3	72° 35' 20.4"	20° 35' 10.8"	403
	P1210011	11.8±0.1	11.9±0.1	3	−36.0 to −31.1	4.3 to 5.0	72° 35' 18.9"	20° 35' 11.0"	403
	P1210012	11.8±0.1		1	−34.6 to −30.2	4.6 to 5.3	72° 35' 20.4"	20° 35' 10.8"	403
	P1210014	10.9±0.1	17.5±0.7	3	−33.2 to −26.8	4.6 to 5.6	72° 35' 18.9"	20° 35' 11.1"	403
*PR4*									
	P1201017	9.61±0.04	12.3±0.2	4	−41.3 to −26.2	3.6 to 6.4	72° 34' 03.8"	20° 52' 21.0"	391
	P1210018	12.9±1.0	14.4±0.6	3	−38.6 to −28.7	4.3 to 6.4	72° 34' 02.2"	20° 52' 09.3"	391
*PR5*									
	P1210032	3.3±0.4		1	−40.0 to −33.4	3.6 to 4.1	71° 59' 12.4"	20° 28' 40.5"	393
	P1210036	7.1±0.1	15.9±4.2	4	−40.3 to −33.5	4.1 to 4.3	71° 59' 12.6"	20° 28' 41.3"	393
*Hola*									
	Hola	6.1±1.6	11.3±0.6	11	−38.0 to −26.7	2.5 to 3.7	68° 55' 05.8"	14° 17' 02.6"	218

VPDB, Vienna Pee Dee Belemnite.
