# Supplementary material for: Timescales of methane seepage on the Norwegian margin following collapse of the Scandinavian Ice Sheet
Source: Nat Commun. 2016 May 11;7:11509. doi: 10.1038/ncomms11509 (PMC4865861; doi:10.1038/ncomms11509)
Supplement: Supplementary Information — Supplementary Figures 1-3, Supplementary Tables 1-3, Supplementary Note 1 and Supplementary References. [file ncomms11509-s1.pdf]

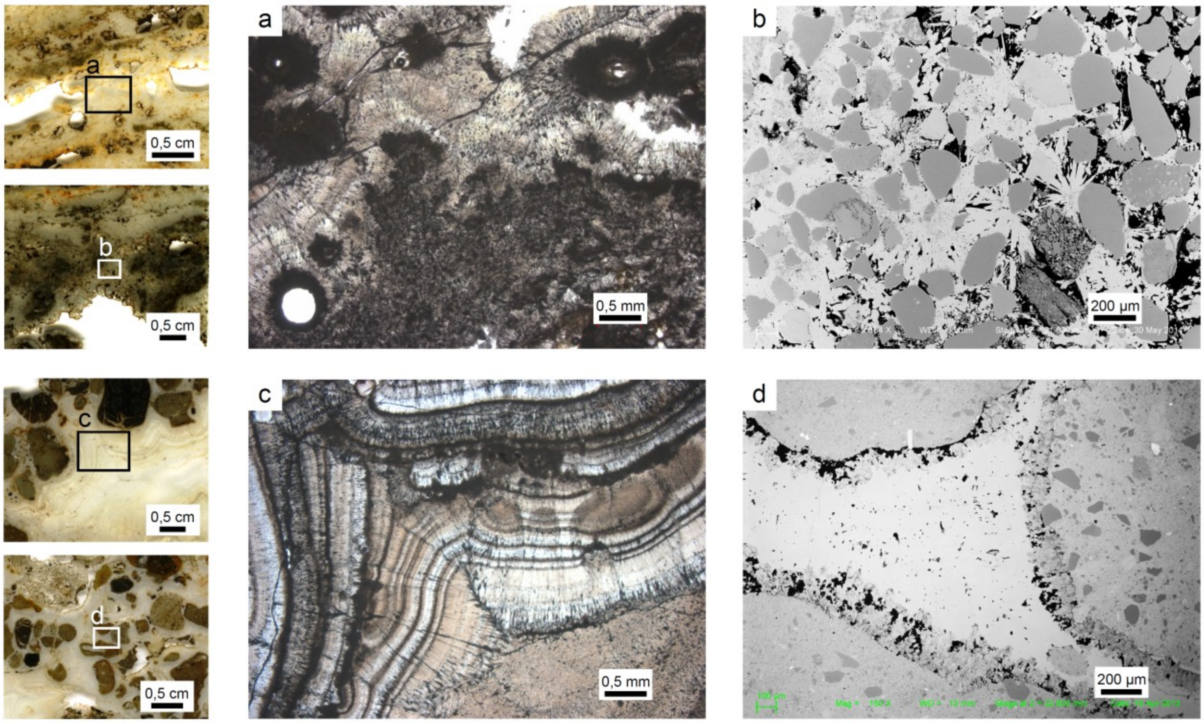

Supplementary Figure 1: Petrographic characteristics of carbonate samples. Micrographs (natural light) of sample P1210002 (a) and P1210017 (c). Scanning electron microscopy (SEM) micrographs of sample P1210002 (b) and P1210017 (d). The early generation carbonates that occur as cements filling the pore space of detrital sediments consist mainly of microcrystalline aragonite (a, b) and minor intragranular high Mg-calcite (d). The early generation carbonate cemented sediments (crusts) are occasionally fragmented into mm- to cm-scale clasts due to current reworking that can form gravelly sediments that in turn can be lithified by aragonite cementation (d). The late stage carbonate precipitation occurs in cavities within crusts that are formed due fluid flushing. The cavities are filled by almost pure acicular to radial fibrous aragonite (a, c), typically forming concentric layers reflecting several episodes of precipitation (c).

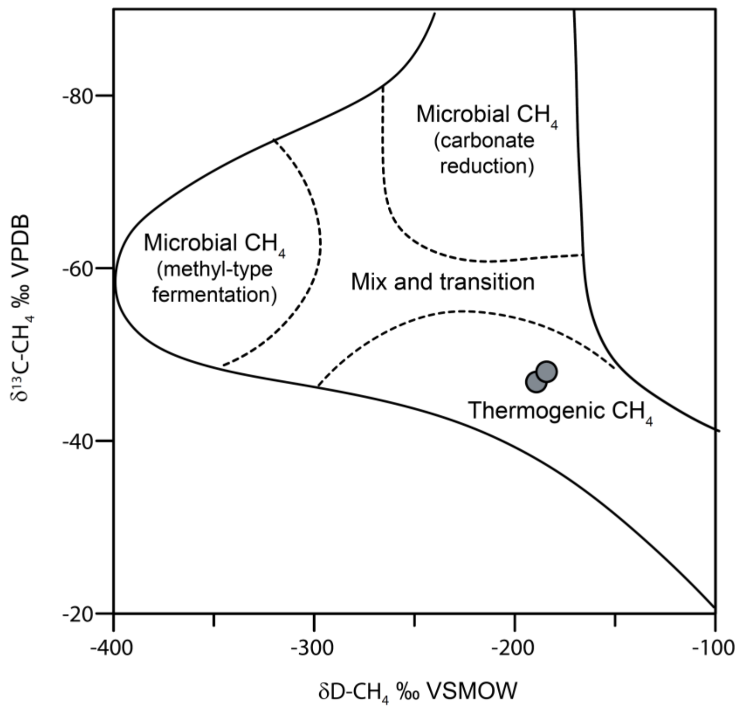

Supplementary Figure 2: Methane stable hydrogen and carbon isotopic compositions of gas samples from the Barents Sea. The fields of microbial and thermogenic methane are defined after [1].

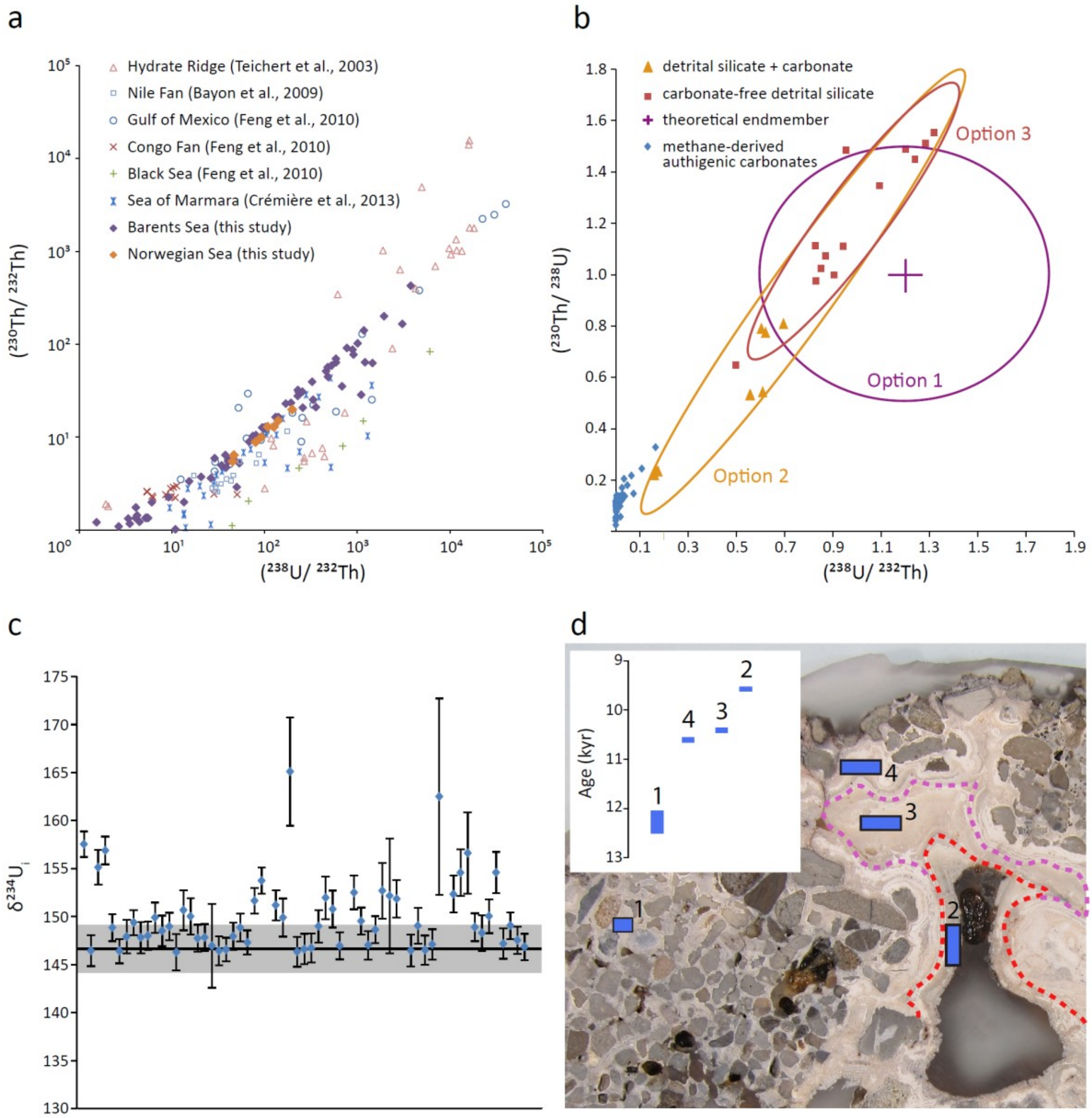

Supplementary Figure 3: (a) Comparison of (^{230}Th/^{232}Th) and (^{238}U/^{232}Th) values between samples from this study, and other occurrences of methane related authigenic carbonates. Note that low (^{230}Th/^{232}Th) and (^{238}U/^{232}Th) values (< 10) correspond to samples that contain a significant proportion of detrital material, while high values (> 100) indicate samples that are largely insensitive to detrital Th and U corrections. (b) Summary plot of authigenic carbonate and detritus analyses illustrating different options for detrital Th and U correction. Each U-Th age from this study represents a two-point model age based on an isochron projected through a measured carbonate composition, and a measured or assumed detritus composition. Because measured detrital activity ratios are highly correlated, they provide a narrower domain through which to project isochron lines compared to the theoretical endmember, and thus result in lower age uncertainties. (c) Comparison between calculated initial δ^{234}U values (errors bars are 2σ) from this study and a mean seawater value of 146.6 ± 2.5 ‰ [2]. (d) U-Th dating results from sample P1210017, showing that detritus-corrected ages are consistent with sample morphology, with matrix-forming carbonate (subsample 1), older than late-stage aragonitic cavity fills. Dotted lines represent the boundaries between different generations of late-stage aragonite, which revealed a resolvable growth history on the order of ~ 1 kyr, with subsample 4 older than subsample 3, and respectively 2.

Supplementary Table 1. Mineralogy of carbonate samples.

Sample	Area	Carbonate minerals	Detrital minerals
P1210001	PR1	aragonite	quartz, plagioclase, illite/muscovite, chlorite
P1210002	PR1	aragonite	quartz, plagioclase, kaolinite
P1210004	PR1	aragonite, high-Mg calcite (13 % mol $MgCO_3$)	quartz, plagioclase, illite/muscovite, chlorite
P1210006	PR1	aragonite	quartz, plagioclase, kaolinite
P1210007	PR1	aragonite	quartz, plagioclase, illite/muscovite, amphibolite
P1210010	PR3	aragonite, high-Mg calcite (14 % mol $MgCO_3$)	quartz, plagioclase, chlorite
P1210011	PR3	aragonite	quartz, plagioclase, illite/muscovite
P12100012	PR3	aragonite	quartz, plagioclase, illite/muscovite, amphibolite
P1210014	PR3	aragonite, high-Mg calcite (14 % mol $MgCO_3$)	quartz, plagioclase, illite/muscovite, chlorite
P1210017	PR4	aragonite, high-Mg calcite (15 % mol $MgCO_3$)	quartz, plagioclase, illite/muscovite, chlorite
P12100018	PR4	aragonite	quartz, illite/muscovite
P1210032	PR5	aragonite	quartz, plagioclase, illite/muscovite, amphibolite, chlorite
P1210036	PR5	aragonite, calcite	quartz, plagioclase, illite/muscovite, amphibolite, chlorite
Hola	Hola	aragonite	quartz, plagioclase, illite/muscovite

Supplementary Table 2. Detrital correction options.

	**($^{232}Th/^{238}U$)**	**± 2σ (%)**	**($^{230}Th/^{238}U$)**	**± 2σ (%)**	**($^{234}U/^{238}U$)**	**± 2σ (%)**	**ρ(2/8)-(0/8)**	**ρ(2/8)-(4/8)**	**ρ(0/8)-(4/8)**
Option 1	1.20000	50.00	1.00000	50.00	1.00000	50.00	0.000	0.000	0.000
Option 2	0.77874	87.32	0.93673	91.15	1.00404	11.57	0.971	-0.964	-0.943
Option 3	0.98294	45.61	1.20642	44.45	0.96748	6.34	0.927	-0.860	-0.781

Supplementary Table 3. Variations of temperature and relative pressure conditions used for gas hydrate stability modelling. The seafloor reference 350 m, is an average of the present-day water depth in the southwest Barents Sea. Note that for the LGM, sea-level and geoid do not account for seafloor pressure as the studied areas were ice grounded.

Parameter	LGM	Ice-sheet collapse: ~18 to 16 ka	Eustatic rebound: ~16 to 9 ka	Present-day: ~9 ka-now	Unit
Ice-sheet thickness[3]	1100	0	0	0	m
Sea level[4]	-	-100	-100 to 0	0	m
Geoid[5]	-	-90	-90 to 0	0	mbsf
Pressure at seafloor	99	34	34 to 35	35	bar
Temperature at seafloor[6]	1	3.5	5	6	°C

Supplementary Note

Carbonate crust samples contained 0.4 – 10.1 ppm of U, and 4 – 7000 ppb of Th. ($^{230}Th/^{232}Th$) activity ratios were between 1 – 427 (Supplementary Data 1), spanning a range of values similar to those reported from other occurrences of methane-related authigenic carbonates (Supplementary Figure 3a,[7–10]). U-Th age calculations were performed in Isoplot v. 3.75 [11], using the decay constants of [12]. As the analysed samples consisted of mixtures of authigenic carbonate (ca. 99 – 25 %) and detrital material, accurate interpretation of their U-Th age requires that measured isotope ratios be corrected for the U and Th isotopic composition of the incorporated detritus. This can be achieved using either a theoretical endmember composition [9,10,13,14], or locally measured detritus compositions [7,8,10,15] . Theoretical endmember compositions typically assume secular equilibrium in the ^{238}U decay chain (i.e. ($^{230}Th/^{238}U$) = ($^{234}U/^{238}U$) = 1), and a ($^{232}Th/^{238}U$) activity ratio close to average values for the upper continental crust or average shale composition (1.2 – 1.33,[16]), with uncertainties arbitrarily set at ± 50%. However, measured detrital ($^{234}U/^{238}U$) and ($^{230}Th/^{238}U$) may deviate from secular equilibrium values due to loss of ^{234}U through alpha-recoil, and adsorbtion of excess ^{230}Th from seawater. Additionally, the presence of methane-related or biogenic carbonate, which preferentially incorporates U relative to Th, would shift ($^{232}Th/^{238}U$) and ($^{230}Th/^{238}U$) activity ratios to values below 1, and ($^{234}U/^{238}U$) towards average seawater composition (1.147 ± 0.003,[2]). This study explored three options for the isotope composition of the detrital material used to correct U-Th ages (Supplementary Table 2). The first of these is a theoretical endmember composition with ($^{232}Th/^{238}U$) = 1.2, ($^{230}Th/^{238}U$) = ($^{234}U/^{238}U$) = 1, and uncertainties set at ± 50 % (2σ). The second option is based on an average detrital composition calculated using data from seven detritus samples. Replicate analyses from each detritus sample (n = 3 – 7) formed distinct clusters (Supplementary Figure 3b), with 3 analyses identified as outliers on the basis of their

relatively high Th-content, suggesting the presence of a Th-rich mineral, such as monazite. Activity ratios varied over a wide range between samples, with ($^{232}Th/^{238}U$) between 0.16-1.31, ($^{230}Th/^{238}U$) between 0.21-1.86, and ($^{234}U/^{238}U$) between 0.92-1.12. Using a mean detrital isotope composition calculated on the basis of all measured detritus analyses to correct carbonate data results in U-Th dates that are 0.5-25 % younger, and ca. 50 % more precise than, but still statistically equivalent to option 1. The lower uncertainties are due to the fact that although the calculated uncertainty of the ($^{232}Th/^{238}U$) and ($^{230}Th/^{238}U$) approaches ± 100 % (2σ), variations in measured activity ratios are highly correlated (ρ($^{232}Th/^{238}U$)-($^{230}Th/^{238}U$) = 0.97, ρ($^{232}Th/^{238}U$) – ($^{234}Th/^{238}U$) = - 0.96, and ρ($^{230}Th/^{238}U$) – ($^{234}Th/^{238}U$) = - 0.94). However, three of the analysed detritus samples (P1210027 6-8 cm, P1210027 13-16 cm, and P1210020 10-13 cm) were estimated to contain up to 6 % carbonate based on weighing HNO_3-insoluble residues during sample preparation, and were characterized by ($^{234}U/^{238}U$) > 1. The implication is that data from these three samples are not suitable to be included in the detrital correction of carbonate crust analyses, because the age of the carbonate fraction is not known, and its presence violates the assumption that crust samples consist of a two-component mixture of methane-related carbonate and detrital silicate. Consequently our third, and preferred option is based on an average isotope composition calculated using only those detritus analyses that are assumed to be free of carbonate, as evidenced by ($^{234}U/^{238}U$) ≤ 1. It should be noted that the differences in U-Th age and its precision between options 2 and 3 are negligible (Supplementary Data 2).

Calculated initial $\delta^{234}U$ values ranged between 146.3 – 165.1 (Supplementary Figure 3c), with 75 % of the samples giving values statistically equivalent to average seawater composition at 146.6 ± 2.5 ‰ [2]. Where multiple subsamples were analysed from the same carbonate crust block, detrital corrected ages were typically consistent with sample morphology. Carbonates cementing sand-gravel size particles were older than late-stage

aragonitic cavity fills, and 0.2 – 2 cm thick layered cavity fills showed resolvable growth histories on the order of 1 kyr (Supplementary Figure 3d).

Supplementary References:

1. Whiticar, M. J. Carbon and hydrogen isotope systematics of bacterial formation and oxidation of methane. *Chem. Geol.* **161,** 291–314 (1999).
2. Robinson, L. F., Belshaw, N. S. & Henderson, G. M. U and Th concentrations and isotope ratios in modern carbonates and waters from the Bahamas. *Geochim. Cosmochim. Acta* **68,** 1777–1789 (2004).
3. Siegert, M. J. & Dowdeswell, J. A. Numerical reconstructions of the Eurasian Ice Sheet and climate during the Late Weichselian. *Quat. Sci. Rev.* **23,** 1273–1283 (2004).
4. Lambeck, K. & Chappell, J. Sea Level Change Through the Last Glacial Cycle. *Science* **292,** 679–686 (2001).
5. Fjeldskaar, W. The Amplitude and Decay of the Glacial Forebulge in Fennoscandia. *Nor. Geol. Tidsskr.* **74,** 2–8 (1994).
6. Aagaard-Sørensen, S., Husum, K., Hald, M. & Knies, J. Paleoceanographic development in the SW Barents Sea during the Late Weichselian–Early Holocene transition. *Quat. Sci. Rev.* **29,** 3442–3456 (2010).
7. Teichert, B. M. A. *et al.* U/Th systematics and ages of authigenic carbonates from Hydrate Ridge, Cascadia Margin: recorders of fluid flow variations. *Geochim. Cosmochim. Acta* **67,** 3845–3857 (2003).
8. Bayon, G., Henderson, G. M. & Bohn, M. U–Th stratigraphy of a cold seep carbonate crust. *Chem. Geol.* **260,** 47–56 (2009).
9. Feng, D. *et al.* U/Th dating of cold-seep carbonates: An initial comparison. *Deep Sea Res. Part II Top. Stud. Oceanogr.* **57,** 2055–2060 (2010).
10. Crémière, A., Bayon, G., Ponzevera, E. & Pierre, C. Paleo-environmental controls on cold seep carbonate authigenesis in the Sea of Marmara. *Earth Planet. Sci. Lett.* **376,** 200–211 (2013).

11. Ludwig, K. R. *User's Manual for Isoplot 3.75: A Geochronological Toolkit for Microsoft Excel*. **5,** (Kenneth R. Ludwig, 2003).

12. Cheng, H. *et al.* Improvements in 230Th dating, 230Th and 234U half-life values, and U–Th isotopic measurements by multi-collector inductively coupled plasma mass spectrometry. *Earth Planet. Sci. Lett.* **371–372,** 82–91 (2013).

13. Bayon, G. *et al.* Formation of carbonate chimneys in the Mediterranean Sea linked to deep-water oxygen depletion. *Nat. Geosci.* **6,** 755–760 (2013).

14. Liebetrau, V. *et al.* Cold-seep-driven carbonate deposits at the Central American forearc: contrasting evolution and timing in escarpment and mound settings. *Int. J. Earth Sci.* 1–28 (2014). doi:10.1007/s00531-014-1045-2

15. Watanabe, Y., Nakai, S. 'ichi, Hiruta, A., Matsumoto, R. & Yoshida, K. U–Th dating of carbonate nodules from methane seeps off Joetsu, Eastern Margin of Japan Sea. *Earth Planet. Sci. Lett.* **272,** 89–96 (2008).

16. Hans Wedepohl, K. The composition of the continental crust. *Geochim. Cosmochim. Acta* **59,** 1217–1232 (1995).
