# Supplementary material for: Timescales of methane seepage on the Norwegian margin following collapse of the Scandinavian Ice Sheet
Source: Nat Commun. 2016 May 11;7:11509. doi: 10.1038/ncomms11509 (PMC4865861; doi:10.1038/ncomms11509)
Supplement: Supplementary Data 1 — U-Th isotopic data [file ncomms11509-s2.doc]

**Supplementary Data 1. U–Th isotopic data. Measured activity ratios are corrected for mass fractionation and spike contributions.**

| **Area** | **Sample** | | **Weight (mg)** | **^238^U (ppm)** | **^232^Th (ppm)** | **(^230^Th/^232^Th)** | **Measured activity ratios** | | | | | |
| --- | --- | --- | --- | --- | --- | --- | --- | --- | --- | --- | --- | --- |
|  |  |  |  |  |  |  | **(^232^Th/^238^U)** | **± 2σ (%)** | **(^230^Th/^238^U)** | **± 2σ (%)** | **(^234^U/^238^U)** | **± 2σ (%)** |
| PR1 |  | |  |  |  |  |  |  |  |  |  |  |
|  | P1210001 1 | | 5.79 | 8.7 | 0.116 | 32.1 | 0.004385 | 0.20 | 0.14058 | 0.42 | 1.1508 | 0.11 |
|  | P1210001 2 | | 4.99 | 8.5 | 0.033 | 91.1 | 0.001293 | 0.18 | 0.11775 | 0.33 | 1.1415 | 0.14 |
|  | P1210001 3 | | 5.52 | 10.1 | 0.798 | 4.7 | 0.025922 | 0.13 | 0.12227 | 0.68 | 1.1464 | 0.11 |
|  | P1210001 4 | | 23.56 | 6.4 | 0.259 | 10.2 | 0.013405 | 0.18 | 0.13734 | 0.40 | 1.1491 | 0.11 |
|  | P1210001 5 | | 12.62 | 4.4 | 0.069 | 23.3 | 0.005203 | 0.18 | 0.12127 | 0.31 | 1.1431 | 0.12 |
|  | P1210001 6 | | 19.13 | 6.1 | 0.005 | 426.6 | 0.000264 | 0.19 | 0.11265 | 0.31 | 1.1418 | 0.11 |
|  | P1210001 7 | | 13.92 | 5.2 | 0.388 | 5.5 | 0.024752 | 0.18 | 0.13490 | 0.32 | 1.1391 | 0.11 |
|  | P1210001 8 | | 14.73 | 8.8 | 0.190 | 16.4 | 0.007094 | 0.18 | 0.11637 | 0.31 | 1.1436 | 0.11 |
|  | P1210002 1 | | 20.28 | 3.4 | 0.045 | 27.7 | 0.004393 | 0.04 | 0.12152 | 0.52 | 1.1422 | 0.14 |
|  | P1210002 2 | | 26.43 | 4.7 | 0.062 | 30.6 | 0.004293 | 0.04 | 0.13151 | 0.44 | 1.1420 | 0.12 |
|  | P1210002 3 | | 24.46 | 4.3 | 0.100 | 16.4 | 0.007605 | 0.04 | 0.12494 | 0.40 | 1.1437 | 0.13 |
|  | P1210002 4 | | 19.93 | 7.0 | 0.264 | 10.5 | 0.012381 | 0.03 | 0.12989 | 0.35 | 1.1415 | 0.13 |
|  | P1210002 5 | | 31.3 | 4.6 | 0.083 | 22.9 | 0.005909 | 0.03 | 0.13512 | 0.29 | 1.1426 | 0.12 |
|  | P1210002 6 | | 24.49 | 5.6 | 0.417 | 5.7 | 0.024669 | 0.04 | 0.14080 | 0.38 | 1.1373 | 0.13 |
|  | P1210002 7 | | 19.83 | 3.8 | 0.338 | 5.0 | 0.029160 | 0.03 | 0.14479 | 0.41 | 1.1407 | 0.13 |
|  | P1210004 1 | | 17.6 | 3.3 | 0.214 | 6.4 | 0.021565 | 0.03 | 0.13831 | 0.43 | 1.1414 | 0.13 |
|  | P1210004 2 | | 34.54 | 7.3 | 0.046 | 44.6 | 0.002069 | 0.04 | 0.092326 | 0.35 | 1.1437 | 0.12 |
|  | P1210004 3 | | 20.7 | 8.5 | 0.026 | 102.2 | 0.000991 | 0.07 | 0.10125 | 0.34 | 1.1436 | 0.12 |
|  | P1210006 1 | | 15.84 | 6.6 | 0.016 | 63.8 | 0.000823 | 0.25 | 0.052489 | 0.39 | 1.1448 | 0.38 |
|  | P1210006 2 | | 13.45 | 5.2 | 0.011 | 62.5 | 0.000686 | 0.18 | 0.042898 | 0.35 | 1.1447 | 0.13 |
|  | P1210006 3 | | 10.59 | 6.5 | 0.029 | 35.2 | 0.001461 | 0.18 | 0.051471 | 0.40 | 1.1443 | 0.11 |
|  | P1210006 4 | | 4 | 6.1 | 0.052 | 21.0 | 0.002801 | 0.14 | 0.058800 | 0.55 | 1.1452 | 0.12 |
|  | P1210006 5 | | 3.64 | 4.3 | 0.004 | 165.1 | 0.000326 | 0.20 | 0.053901 | 0.63 | 1.1466 | 0.12 |
|  | P1210006 6 | | 4.43 | 9.5 | 0.026 | 28.5 | 0.000906 | 0.18 | 0.025821 | 0.68 | 1.1462 | 0.11 |
|  | P1210007 1 | | 3.44 | 8.6 | 0.029 | 77.4 | 0.001096 | 0.19 | 0.084821 | 0.53 | 1.1480 | 0.11 |
|  | P1210007 2 | | 5.21 | 5.9 | 0.039 | 51.3 | 0.002171 | 0.18 | 0.11138 | 0.35 | 1.1487 | 0.11 |
| PR3 |  | |  |  |  |  |  |  |  |  |  |  |
|  | P1210010 1 | | 16.83 | 2.4 | 0.015 | 56.5 | 0.002086 | 0.07 | 0.11789 | 0.59 | 1.1460 | 0.13 |
|  | P1210010 2 | | 17.83 | 2.3 | 0.027 | 30.7 | 0.003913 | 0.29 | 0.12024 | 0.87 | 1.1444 | 0.17 |
|  | P1210010 3 | | 3.66 | 0.4 | 0.074 | 3.3 | 0.065227 | 0.46 | 0.21800 | 0.82 | 1.1457 | 0.34 |
|  | P1210011 1 | | 6.6 | 4.4 | 0.023 | 69.8 | 0.001700 | 0.18 | 0.11867 | 0.37 | 1.1413 | 0.13 |
|  | P1210011 2 | | 5.22 | 2.6 | 0.007 | 140.6 | 0.000849 | 0.18 | 0.11945 | 0.46 | 1.1416 | 0.13 |
|  | P1210011 3 | | 10.29 | 4.1 | 0.127 | 12.5 | 0.010295 | 0.18 | 0.12901 | 0.32 | 1.1401 | 0.12 |
|  | P1210012 1 | | 6.27 | 2.8 | 0.026 | 39.4 | 0.003069 | 0.44 | 0.12077 | 0.52 | 1.1436 | 0.14 |
|  | P1210014 1 | | 47.49 | 4.4 | 0.464 | 5.9 | 0.034613 | 0.28 | 0.20509 | 0.68 | 1.1385 | 0.12 |
|  | P1210014 2 | | 14.32 | 2.1 | 0.092 | 8.9 | 0.014721 | 0.08 | 0.13119 | 0.88 | 1.1433 | 0.15 |
|  | P1210014 3 | | 23.14 | 5.2 | 0.030 | 58.8 | 0.001895 | 0.06 | 0.11137 | 0.42 | 1.1422 | 0.12 |
| PR4 |  | |  |  |  |  |  |  |  |  |  |  |
|  | P1210017 1 | | 6.85 | 5.4 | 0.174 | 12.6 | 0.010609 | 0.06 | 0.13410 | 0.57 | 1.1454 | 0.14 |
|  | P1210017 2 | | 48.14 | 6.3 | 0.022 | 87.3 | 0.001123 | 0.06 | 0.098048 | 0.31 | 1.1453 | 0.12 |
|  | P1210017 3 | | 34.96 | 6.3 | 0.010 | 200.2 | 0.000514 | 0.06 | 0.10281 | 0.35 | 1.1428 | 0.13 |
|  | P1210017 4 | | 64.34 | 6.2 | 0.032 | 63.6 | 0.001697 | 0.06 | 0.10790 | 0.29 | 1.1440 | 0.12 |
|  | P1210018 3 | | 15.73 | 3.5 | 0.521 | 3.7 | 0.048465 | 0.06 | 0.18044 | 0.46 | 1.1385 | 0.13 |
|  | P1210018 4 | | 17.61 | 5.6 | 1.842 | 2.3 | 0.108683 | 0.06 | 0.24508 | 0.35 | 1.1271 | 0.12 |
|  | P1210018 5 | | 21.72 | 5.7 | 0.463 | 6.4 | 0.026528 | 0.06 | 0.17005 | 0.31 | 1.1411 | 0.12 |
| PR5 |  | |  |  |  |  |  |  |  |  |  |  |
|  | P1210032 1 | | 6.61 | 6.8 | 0.411 | 2.9 | 0.019984 | 0.20 | 0.057724 | 0.95 | 1.1415 | 0.12 |
|  | P1210036 1 | | 11.79 | 6.0 | 0.340 | 5.2 | 0.018778 | 0.07 | 0.09828 | 0.64 | 1.1425 | 0.14 |
|  | P1210036 2 | | 15.84 | 6.7 | 0.061 | 25.3 | 0.002996 | 0.11 | 0.075723 | 0.84 | 1.1431 | 0.13 |
|  | P1210036 3 | | 14.91 | 4.6 | 0.054 | 20.7 | 0.003877 | 0.06 | 0.080268 | 0.71 | 1.1433 | 0.13 |
|  | P1210036 4 | | 3.71 | 3.2 | 1.617 | 2.0 | 0.165405 | 0.20 | 0.32841 | 1.28 | 1.1242 | 0.13 |
| Hola |  | |  |  |  |  |  |  |  |  |  |  |
|  | Hola 1 | | 6.96 | 2.8 | 0.184 | 5.7 | 0.021536 | 0.04 | 0.12373 | 0.51 | 1.1442 | 0.13 |
|  | Hola 2 | | 9.69 | 3.1 | 0.363 | 3.6 | 0.038057 | 0.06 | 0.13776 | 1.85 | 1.1435 | 0.12 |
|  | Hola 3 | | 10.38 | 2.3 | 0.511 | 2.0 | 0.074126 | 0.04 | 0.14773 | 0.72 | 1.1400 | 0.13 |
|  | Hola 4 | | 75.05 | 2.5 | 0.097 | 8.8 | 0.012533 | 0.22 | 0.11074 | 0.78 | 1.1427 | 0.11 |
|  | Hola 5 | | 26.7 | 2.2 | 0.147 | 5.5 | 0.022130 | 0.15 | 0.12110 | 1.44 | 1.1404 | 0.13 |
|  | Hola 6 | | 51.42 | 2.5 | 0.161 | 6.4 | 0.021394 | 1.06 | 0.13650 | 3.01 | 1.1415 | 0.12 |
|  | Hola 7 | | 2.49 | 2.6 | 0.085 | 9.9 | 0.010918 | 0.20 | 0.10861 | 0.69 | 1.1485 | 0.18 |
|  | Hola 8 | | 6.78 | 2.6 | 0.056 | 15.1 | 0.007124 | 0.20 | 0.10734 | 0.63 | 1.1419 | 0.13 |
|  | Hola 9 | | 31.78 | 2.3 | 0.064 | 12.9 | 0.009240 | 0.20 | 0.11926 | 0.39 | 1.1429 | 0.11 |
|  | Hola 10 | | 15.33 | 2.7 | 0.066 | 12.9 | 0.007941 | 0.20 | 0.10237 | 0.45 | 1.14230 | 0.12 |
|  | Hola 11 | | 13.11 | 2.7 | 0.041 | 19.8 | 0.005037 | 0.21 | 0.099589 | 0.76 | 1.14214 | 0.11 |
| Rejected samples | | |  |  |  |  |  |  |  |  |  |  |
|  | Hola 12 | | 81 | 7.2 | 7.069 | 0.8 | 0.322515 | 1062.25 | 0.27034 | 1062.25 | 0.27543 | 105.81 |
|  | Hola 13 | | 5.89 | 3.5 | 3.039 | 1.2 | 0.285480 | 0.04 | 0.33267 | 0.45 | 1.1102 | 0.14 |
|  | Hola 14 | | 5.04 | 2.5 | 2.880 | 1.1 | 0.376239 | 0.04 | 0.41088 | 0.34 | 1.1012 | 0.14 |
|  | Hola 15 | | 6.64 | 3.4 | 2.529 | 1.3 | 0.246158 | 0.04 | 0.33125 | 0.29 | 1.1123 | 0.13 |
|  | Hola 16 | | 5.75 | 2.7 | 1.951 | 1.4 | 0.238337 | 0.04 | 0.34194 | 0.37 | 1.1210 | 0.15 |
|  | Hola 17 | | 5.37 | 4.4 | 2.600 | 1.3 | 0.192594 | 0.04 | 0.25888 | 0.33 | 1.1197 | 0.13 |
|  | Hola 18 | | 6.53 | 4.8 | 2.646 | 1.3 | 0.181156 | 1.23 | 0.24340 | 1.47 | 1.0933 | 1.58 |
|  | Hola 19 | | 7.04 | 4.6 | 3.147 | 1.2 | 0.224393 | 0.04 | 0.27529 | 0.29 | 1.1191 | 0.12 |
|  | P1210018 1 | | 55.41 | 3.6 | 3.227 | 1.3 | 0.297169 | 0.08 | 0.39383 | 0.30 | 1.1032 | 0.12 |
|  | P1210018 2 | | 64.25 | 4.1 | 3.048 | 1.7 | 0.244976 | 0.09 | 0.42835 | 0.28 | 1.1226 | 0.11 |
|  | P1210018 6 | | 29.76 | 4.8 | 2.783 | 1.4 | 0.188779 | 0.06 | 0.25652 | 0.29 | 1.1222 | 0.12 |
|  | P1210020 1 | | 3.94 | 1.3 | 4.624 | 0.9 | 1.148640 | 0.09 | 1.0520 | 0.63 | 0.99917 | 0.24 |
|  | P1210020 2 | | 5.75 | 1.2 | 4.579 | 0.9 | 1.286390 | 0.09 | 1.1823 | 0.55 | 0.96856 | 0.24 |
|  | P1210020 3 | | 10.22 | 1.2 | 4.911 | 0.9 | 1.403146 | 0.08 | 1.2118 | 0.37 | 0.95655 | 0.18 |
|  | P1210020 4 | | 14.14 | 2.5 | 7.107 | 0.8 | 0.928265 | 0.08 | 0.69997 | 0.34 | 1.0400 | 0.14 |
|  | P1210032 2 | | 3.76 | 5.2 | 1.455 | 1.0 | 0.092041 | 0.20 | 0.093582 | 0.69 | 1.1423 | 0.15 |
|  | P1210035 1 | | 15.09 | 1.5 | 2.918 | 1.2 | 0.653368 | 0.20 | 0.78958 | 0.43 | 1.0723 | 0.15 |
| Detritus samples | | | |  |  |  |  |  |  |  |  |  |
| P1210006 A | | 33.9 | | 1.6 | 4.508 | 1.2 | 0.941540 | 0.07 | 1.1111 | 0.28 | 0.98212 | 0.14 |
| P1210006 B | | 32.7 | | 1.4 | 3.425 | 1.3 | 0.827797 | 0.07 | 1.1134 | 0.28 | 0.98025 | 0.14 |
| P1210006 C | | 35.7 | | 1.8 | 4.742 | 1.2 | 0.871134 | 0.07 | 1.0728 | 0.27 | 1.0040 | 0.13 |
| P1210006 D | | 42.9 | | 1.7 | 4.773 | 1.2 | 0.942414 | 0.09 | 1.1115 | 0.34 | 0.97933 | 0.13 |
| P1210006 E | | 43.8 | | 2.5 | 13.083 | 0.9 | 1.693103 | 0.09 | 1.5542 | 0.28 | 1.0401 | 0.12 |
| P1210006 F | | 67.2 | | 2.1 | 3.144 | 1.3 | 0.499021 | 0.06 | 0.64775 | 0.26 | 0.99629 | 0.12 |
| P1210028 A | | 96.66 | | 1.4 | 4.006 | 1.6 | 0.95446 | 1.44 | 1.4840 | 2.98 | 0.97795 | 0.12 |
| P1210028 B | | 94.87 | | 1.5 | 4.998 | 1.2 | 1.0935 | 2.88 | 1.3463 | 3.66 | 0.98443 | 0.12 |
| P1210028 C | | 56.9 | | 1.4 | 746.0 | 0.8 | 170.58 | 4.71 | 140.74 | 4.70 | 0.97870 | 0.13 |
| P1210027 6-8 A | | 130.23 | | 1.7 | 3.531 | 1.2 | 0.69558 | 0.23 | 0.80696 | 0.42 | 1.0216 | 0.11 |
| P1210027 6-8 B | | 98.26 | | 1.6 | 2.960 | 1.3 | 0.60380 | 0.60 | 0.78812 | 0.90 | 1.0280 | 0.11 |
| P1210027 6-8 C | | 138.06 | | 1.6 | 3.068 | 1.2 | 0.62133 | 0.31 | 0.77276 | 0.51 | 1.0260 | 0.11 |
| P1210027 13-16 A | | 61.99 | | 9.7 | 4.767 | 1.4 | 0.16137 | 0.32 | 0.21839 | 0.45 | 1.1165 | 0.11 |
| P1210027 13-16 B | | 87.71 | | 8.2 | 4.193 | 1.4 | 0.16825 | 0.20 | 0.23589 | 0.64 | 1.1167 | 0.11 |
| P1210027 13-16 C | | 67.49 | | 9.0 | 4.840 | 1.3 | 0.17583 | 1.36 | 0.23275 | 1.56 | 1.1156 | 0.11 |
| P1210020 0-2 A | | 92.15 | | 2.5 | 6.827 | 1.1 | 0.90416 | 0.22 | 0.99917 | 0.45 | 0.98295 | 0.11 |
| P1210020 0-2 B | | 71.18 | | 2.3 | 5.872 | 1.2 | 0.83023 | 0.38 | 0.97570 | 0.81 | 0.98406 | 0.11 |
| P1210020 0-2 C | | 79.2 | | 2.3 | 6.001 | 1.2 | 0.85015 | 4.44 | 1.0251 | 4.72 | 0.98538 | 0.12 |
| P1210020 10-13 A | | 62.91 | | 5.1 | 8.739 | 0.9 | 0.55846 | 0.21 | 0.52945 | 0.48 | 1.0412 | 0.11 |
| P1210020 10-13 B | | 37.39 | | 5.3 | 9.016 | 1.0 | 0.55600 | 0.20 | 0.52923 | 0.37 | 1.0410 | 0.11 |
| P1210020 10-13 C | | 63.14 | | 5.1 | 9.490 | 0.9 | 0.60926 | 0.23 | 0.54148 | 0.59 | 1.0417 | 0.11 |
| P1210014 A | | 40.29 | | 2.0 | 7.469 | 1.2 | 1.2407 | 0.05 | 1.4496 | 0.26 | 0.92371 | 0.11 |
| P1210014 B | | 77.64 | | 1.9 | 7.606 | 1.2 | 1.2851 | 0.06 | 1.5108 | 0.26 | 0.91894 | 0.11 |
| P1210014 C | | 48.49 | | 1.6 | 6.574 | 1.2 | 1.3199 | 0.08 | 1.5535 | 0.35 | 0.91990 | 0.12 |
| P1210014 D | | 80.29 | | 2.0 | 7.281 | 1.2 | 1.2011 | 0.68 | 1.4892 | 0.84 | 0.92538 | 0.12 |
| P1210014 E | | 47.81 | | 1.9 | 13.685 | 1.2 | 2.3646 | 0.68 | 2.8629 | 0.84 | 0.93122 | 0.11 |
