# Supplementary material for: Timescales of methane seepage on the Norwegian margin following collapse of the Scandinavian Ice Sheet
Source: Nat Commun. 2016 May 11;7:11509. doi: 10.1038/ncomms11509 (PMC4865861; doi:10.1038/ncomms11509)
Supplement: Supplementary Data 2 — U-Th detritus-corrected age [file ncomms11509-s3.doc]

**Supplementary Data 2. U–Th detritus-corrected ages and δ^234^U_i_. Negative values are not shown.**

| **Area** | **Sample** | **Detrital correction,**  **option 1** | | | **Detrital correction,**  **option 2** | | | **Detrital correction,**  **option 3** | | | |
| --- | --- | --- | --- | --- | --- | --- | --- | --- | --- | --- | --- |
|  |  | **Age (ka)** | **± 2σ (abs)** | **Age (ka)** | | **± 2σ (abs)** | **Age (ka)** | | **± 2σ (abs)** | **δ^234^Ui** | **± 2σ (abs)** |
| PR1 |  |  |  |  | |  |  | |  |  |  |
|  | P1210001 1 | 13.82 | 0.25 | 13.67 | | 0.13 | 13.66 | | 0.11 | 157.5 | 1.3 |
|  | P1210001 2 | 11.74 | 0.09 | 11.70 | | 0.06 | 11.70 | | 0.05 | 146.4 | 1.6 |
|  | P1210001 3 | 10.20 | 1.48 | 9.27 | | 0.67 | 9.21 | | 0.54 | 155.2 | 1.8 |
|  | P1210001 4 | 12.78 | 0.76 | 12.31 | | 0.34 | 12.28 | | 0.28 | 156.9 | 1.4 |
|  | P1210001 5 | 11.79 | 0.30 | 11.61 | | 0.14 | 11.59 | | 0.11 | 148.9 | 1.4 |
|  | P1210001 6 | 11.28 | 0.04 | 11.27 | | 0.04 | 11.27 | | 0.04 | 146.4 | 1.3 |
|  | P1210001 7 | 11.73 | 1.42 | 10.84 | | 0.64 | 10.78 | | 0.52 | 147.9 | 1.7 |
|  | P1210001 8 | 11.11 | 0.40 | 10.86 | | 0.18 | 10.85 | | 0.15 | 149.4 | 1.3 |
|  | P1210002 1 | 11.89 | 0.26 | 11.73 | | 0.13 | 11.72 | | 0.11 | 147.8 | 1.6 |
|  | P1210002 2 | 12.97 | 0.25 | 12.82 | | 0.13 | 12.81 | | 0.11 | 148.0 | 1.5 |
|  | P1210002 3 | 11.98 | 0.43 | 11.71 | | 0.20 | 11.70 | | 0.16 | 149.9 | 1.6 |
|  | P1210002 4 | 12.16 | 0.70 | 11.72 | | 0.32 | 11.69 | | 0.26 | 148.5 | 1.6 |
|  | P1210002 5 | 13.22 | 0.34 | 13.01 | | 0.16 | 13.00 | | 0.13 | 149.0 | 1.4 |
|  | P1210002 6 | 12.39 | 1.41 | 11.50 | | 0.64 | 11.45 | | 0.52 | 146.3 | 1.9 |
|  | P1210002 7 | 12.43 | 1.67 | 11.37 | | 0.75 | 11.30 | | 0.61 | 150.7 | 2.0 |
|  | P1210004 1 | 12.33 | 1.23 | 11.55 | | 0.56 | 11.50 | | 0.45 | 150.0 | 1.9 |
|  | P1210004 2 | 9.00 | 0.12 | 8.93 | | 0.06 | 8.92 | | 0.05 | 147.7 | 1.4 |
|  | P1210004 3 | 10.01 | 0.07 | 9.98 | | 0.05 | 9.97 | | 0.04 | 147.8 | 1.4 |
|  | P1210006 1 | 5.05 | 0.05 | 5.02 | | 0.03 | 5.02 | | 0.03 | 146.9 | 4.4 |
|  | P1210006 2 | 4.11 | 0.04 | 4.08 | | 0.02 | 4.08 | | 0.02 | 146.4 | 1.5 |
|  | P1210006 3 | 4.90 | 0.08 | 4.85 | | 0.04 | 4.84 | | 0.04 | 146.6 | 1.2 |
|  | P1210006 4 | 5.52 | 0.16 | 5.42 | | 0.08 | 5.41 | | 0.07 | 148.0 | 1.4 |
|  | P1210006 5 | 5.22 | 0.04 | 5.21 | | 0.04 | 5.21 | | 0.04 | 148.8 | 1.4 |
|  | P1210006 6 | 2.41 | 0.05 | 2.38 | | 0.03 | 2.38 | | 0.03 | 147.3 | 1.3 |
|  | P1210007 1 | 8.27 | 0.08 | 8.23 | | 0.05 | 8.23 | | 0.05 | 151.7 | 1.3 |
|  | P1210007 2 | 10.93 | 0.13 | 10.85 | | 0.07 | 10.85 | | 0.06 | 153.7 | 1.3 |
| PR3 |  |  |  |  | |  |  | |  |  |  |
|  | P1210010 1 | 11.65 | 0.14 | 11.57 | | 0.09 | 11.57 | | 0.09 | 151.2 | 1.6 |
|  | P1210010 2 | 11.77 | 0.25 | 11.63 | | 0.15 | 11.62 | | 0.14 | 149.9 | 2.0 |
|  | P1210010 3 | 17.65 | 3.82 | 15.23 | | 1.75 | 15.07 | | 1.42 | 165.2 | 5.5 |
|  | P1210011 1 | 11.81 | 0.11 | 11.75 | | 0.06 | 11.75 | | 0.06 | 146.3 | 1.6 |
|  | P1210011 2 | 11.96 | 0.08 | 11.93 | | 0.06 | 11.93 | | 0.06 | 146.6 | 1.6 |
|  | P1210011 3 | 12.24 | 0.58 | 11.88 | | 0.26 | 11.85 | | 0.22 | 146.7 | 1.5 |
|  | P1210012 1 | 11.90 | 0.19 | 11.79 | | 0.10 | 11.79 | | 0.09 | 149.0 | 1.7 |
|  | P1210014 1 | 18.79 | 2.00 | 17.54 | | 0.91 | 17.45 | | 0.74 | 152.0 | 2.2 |
|  | P1210014 2 | 12.09 | 0.84 | 11.56 | | 0.39 | 11.53 | | 0.33 | 150.8 | 1.9 |
|  | P1210014 3 | 11.01 | 0.12 | 10.95 | | 0.07 | 10.94 | | 0.06 | 147.0 | 1.4 |
| PR4 |  |  |  |  | |  |  | |  |  |  |
|  | P1210017 1 | 12.70 | 0.60 | 12.33 | | 0.28 | 12.30 | | 0.23 | 152.5 | 1.7 |
|  | P1210017 2 | 9.65 | 0.07 | 9.61 | | 0.04 | 9.61 | | 0.04 | 149.5 | 1.4 |
|  | P1210017 3 | 10.22 | 0.05 | 10.20 | | 0.04 | 10.20 | | 0.04 | 147.0 | 1.5 |
|  | P1210017 4 | 10.65 | 0.10 | 10.59 | | 0.06 | 10.58 | | 0.05 | 148.6 | 1.4 |
|  | P1210018 3 | 14.84 | 2.82 | 13.05 | | 1.28 | 12.93 | | 1.04 | 152.7 | 2.8 |
|  | P1210018 4 | 17.54 | 6.70 | 13.26 | | 3.11 | 12.97 | | 2.52 | 152.3 | 5.7 |
|  | P1210018 5 | 15.42 | 1.52 | 14.46 | | 0.68 | 14.40 | | 0.55 | 151.9 | 1.9 |
| PR5 |  |  |  |  | |  |  | |  |  |  |
|  | P1210032 1 | 4.05 | 1.14 | 3.34 | | 0.51 | 3.29 | | 0.42 | 146.5 | 1.6 |
|  | P1210036 1 | 8.29 | 1.07 | 7.62 | | 0.48 | 7.58 | | 0.39 | 149.1 | 1.8 |
|  | P1210036 2 | 7.23 | 0.18 | 7.12 | | 0.10 | 7.11 | | 0.09 | 146.5 | 1.5 |
|  | P1210036 3 | 7.62 | 0.23 | 7.48 | | 0.11 | 7.47 | | 0.10 | 147.1 | 1.6 |
|  | P1210036 4 | 23.31 | 10.70 | 16.38 | | 5.12 | 15.91 | | 4.17 | 162.7 | 9.8 |
| Hola |  |  |  |  | |  |  | |  |  |  |
|  | Hola 1 | 10.73 | 1.23 | 9.96 | | 0.55 | 9.91 | | 0.45 | 152.4 | 1.9 |
|  | Hola 2 | 10.91 | 2.21 | 9.53 | | 1.03 | 9.43 | | 0.85 | 154.6 | 2.4 |
|  | Hola 3 | 9.04 | 4.40 | 6.25 | | 2.02 | 6.06 | | 1.64 | 156.7 | 4.0 |
|  | Hola 4 | 10.09 | 0.71 | 9.65 | | 0.33 | 9.62 | | 0.27 | 148.9 | 1.4 |
|  | Hola 5 | 10.44 | 1.28 | 9.65 | | 0.60 | 9.60 | | 0.49 | 148.3 | 1.8 |
|  | Hola 6 | 12.14 | 1.30 | 11.38 | | 0.70 | 11.33 | | 0.63 | 150.1 | 1.7 |
|  | Hola 7 | 9.95 | 0.62 | 9.56 | | 0.29 | 9.54 | | 0.24 | 154.6 | 2.2 |
|  | Hola 8 | 10.18 | 0.41 | 9.93 | | 0.19 | 9.91 | | 0.16 | 147.2 | 1.6 |
|  | Hola 9 | 11.26 | 0.52 | 10.93 | | 0.24 | 10.91 | | 0.19 | 149.1 | 1.4 |
|  | Hola 10 | 9.59 | 0.45 | 9.31 | | 0.21 | 9.29 | | 0.17 | 147.5 | 1.4 |
|  | Hola 11 | 9.53 | 0.29 | 9.35 | | 0.15 | 9.34 | | 0.13 | 146.8 | 1.4 |
| Rejected samples | |  |  |  | |  |  | |  |  |  |
|  | Hola 12 | - | - | - | | - | - | | - | - | - |
|  | Hola 13 | 12.51 | 20.94 | - | | 10.67 | - | | 8.70 | 167.5 | 20.2 |
|  | Hola 14 | 14.35 | 30.53 | - | | 16.66 | - | | 13.66 | 180.3 | 33.3 |
|  | Hola 15 | 16.27 | 17.34 | 4.80 | | 8.62 | 3.99 | | 7.02 | 162.4 | 16.0 |
|  | Hola 16 | 18.35 | 16.49 | 7.46 | | 8.16 | 6.70 | | 6.64 | 173.2 | 16.5 |
|  | Hola 17 | 11.77 | 12.85 | 3.39 | | 6.20 | 2.81 | | 5.04 | 158.0 | 11.4 |
|  | Hola 18 | 11.24 | 12.32 | 3.22 | | 5.94 | 2.67 | | 4.83 | 122.6 | 22.9 |
|  | Hola 19 | 10.83 | 15.41 | 0.69 | | 7.56 | - | | - | 163.9 | 14.3 |
|  | P1210018 1 | 20.36 | 22.16 | 5.45 | | 11.38 | 4.38 | | 9.29 | 163.9 | 20.8 |
|  | P1210018 2 | 30.32 | 16.94 | 19.12 | | 8.40 | 18.33 | | 6.84 | 182.8 | 18.2 |
|  | P1210018 6 | 11.80 | 12.52 | 3.64 | | 6.03 | 3.08 | | 4.90 | 160.2 | 11.2 |
|  | P1210020 1 | - | - | - | | - | - | | - | - | - |
|  | P1210020 2 | - | - | 107.25 | | 62.51 | 112.92 | | 47.95 | 2.3 | 210.7 |
|  | P1210020 3 | 522.63 | 3.41 | 523.62 | | 2.37 | 523.69 | | 2.26 | 337.5 | 401.4 |
|  | P1210020 4 | - | - | - | | - | - | | - | - | - |
|  | P1210032 2 | 1.74 | 5.55 | - | | - | - | | 2.07 | 159.5 | 5.2 |
|  | P1210035 1 | 66.94 | 77.36 | 1.40 | | 65.28 | - | | 56.13 | 277.0 | 181.8 |
